# A new approach methodology for studying intrinsic ventricular arrhythmias in Fabry disease

**DOI:** 10.3389/fcvm.2026.1769383

**Published:** 2026-04-23

**Authors:** Andre Monteiro da Rocha, Liming Shu, Prakaimuk Saraithong, Erinn Laimon-Thomson, Kalai Muthukumarasamy, Todd Herron, James A. Shayman

**Affiliations:** 1 Department of Internal Medicine, Cardiology, University of Michigan, Ann Arbor, MI, United States; 2 Department of Internal Medicine, Nephrology, University of Michigan, Ann Arbor, MI, United States

**Keywords:** action potential, cardiac arrhythmia, Fabry disease (FD), α-galactosidase A (GLA), hiPSC-cardiomyocytes, intracellular calcium, new approach methodology (NAM), optical mapping

## Abstract

**Background:**

Sudden cardiac death is a common but poorly understood cause of mortality in Fabry disease (FD). We investigated the arrhythmogenic mechanisms using a new approach methodology model of FD. This model employed optical mapping of membrane potentials and calcium transient (CaT) with adenovirus-mediated replacement of GLA (advGLA) in stem cell-derived ventricular cardiomyocytes (VCMs) of WT and FD origin.

**Methods:**

α-Galactosidase A (GLA) null and wild-type (WT; iPS-DF19-9-11T) stem cells were differentiated into VCMs using a GiWi protocol, selected using MACS, and matured on MatrixPlus-coated 96- or 6-well plates for 7 days. GLA-null, wild-type, and adenoviral-transfected GLA ventricular cardiomyocytes were then subjected to optical mapping to measure membrane potentials using Fluovolt and calcium transients using Calbryte 520AM. In addition, cells underwent poly(A)-enriched RNA sequencing.

**Results:**

Fabry disease-derived matured cardiomyocyte syncytia presented a wide array of arrhythmias, including tachyarrhythmias, early after depolarizations, quiescence, and beat irregularity, compared with wild-type cardiomyocytes. Furthermore, optical mapping of intracellular calcium transients indicated that GLA-null ventricular cardiomyocytes presented an altered frequency of intracellular calcium release, prolonged calcium transient duration at 80% return to baseline, increased calcium transient triangulation, decreased calcium fluorescence upstroke, and higher baseline calcium fluorescence and amplitude relative to wild-type cardiomyocytes. Furthermore, in response to isoproterenol, the relative change in contraction frequency was higher in GLA-null cardiomyocytes compared with wild-type ventricular cardiomyocytes, whereas the change in baseline fluorescence was lower. Transcriptomic analysis indicated that several genes encoding sodium channels subunits (five genes), potassium channel subunits (29 genes), and calcium channels (nine genes) were differentially expressed between GLA-null and wild-type ventricular cardiomyocytes. Adenovirus-mediated rescue of GLA expression (advGLA) did not reduce the frequency of arrhythmias. In addition, treatment of GLA-null cardiomyocytes with advGLA equalized the expression of sodium channel genes (eight genes), potassium channel genes (32 genes), and calcium channel genes (12 genes) relative to wild-type cardiomyocytes.

**Conclusion:**

We developed a new approach methodology for Fabry disease-associated arrhythmias using hiPSC-VCMs, which are characterized by a high frequency of arrhythmias associated with widespread dysregulation of ion channel expression. GLA overexpression was unable to restore appropriate ion channel expression and eliminate arrhythmias.

## Introduction

Fabry disease (FD) is the most prevalent glycosphingolipid storage disorder (1, 2). FD arises from mutations in the X-linked gene, *GLA* (3–6), which encodes the glycoprotein α-galactosidase A (α-Gal A) (3, 4). GLA forms homodimers that hydrolyze terminal α-galactosyl moieties from glycolipids and glycoproteins. Deficiency in α-Gal A results in lysosomal and extra-lysosomal accumulation of glycosphingolipids (GSLs) (7), such as globotriaosylceramide (Gb_3_), globotriaosylsphingosine, and galabiosylceramide (8, 9).

FD is a systemic disorder with clinical manifestations that include acroparesthesias, angiokeratomas, anhidrosis or hypohidrosis, stroke, renal insufficiency that often progresses to renal failure, cardiomyopathy, and cardiac arrhythmias (10–14). Cardiac complications are the primary cause of mortality in FD (15, 16) and account for up to 50% of deaths among FD patients (17). Historically, the cardiac phenotype was regarded as a lysosomal storage cardiomyopathy characterized by left ventricular hypertrophy, leading to diastolic and systolic left ventricular dysfunction (18–21). More recently, arrhythmias and sudden cardiac arrest have been identified as the principal drivers of premature mortality in individuals with FD (15, 22–25). Although FD is an X-linked disorder, heterozygous females may present cardiac alterations similar to those observed in hemizygous males, albeit with phenotypes that manifest later in life (26, 27).

Abnormal glycolipid accumulation has been observed in various types of cardiac cells, including cardiomyocytes, fibroblasts, and Purkinje cells (28, 29). Although cardiac hypertrophy, inflammation, fibrosis, and GSL accumulation have all been associated with arrhythmogenesis in FD, the precise mechanisms underlying arrhythmogenesis remain poorly understood. Importantly, there is little evidence that clearance of GSL accumulation by enzyme replacement therapy (ERT) improves the cardiac manifestation of FD (27). However, there is a general acknowledgement that ERT alleviates renal symptoms in FD patients (27).

New approach methodologies (NAMs) are innovative approaches, strategies, and techniques for modeling and generating relevant data on human physiological states and diseases (30). NAMs comprise *in vitro*, *in chemico*, and *ex vivo* strategies. In pursuit of a NAM suitable for studying arrhythmias in FD, we used human fibroblasts to generate human induced pluripotent stem cells (hiPSCs), which were subsequently differentiated into mature ventricular cardiomyocytes (hiPSC-VCMs). Comparison of the electrophysiological properties and intracellular calcium transient characteristics of FD and wild-type (WT) hiPSC-VCMs revealed a pattern of chaotic arrhythmias intrinsic to FD. Furthermore, adenovirus-mediated restoration of GLA activity failed to prevent the development of complex arrhythmias or to correct abnormalities in the transcription of genes encoding ion channels.

## Methods

### Detection of globotriaosylceramide using high-performance thin-layer chromatography

Gb_3_ measurements using high-performance thin-layer chromatography (HPTLC) were performed as previously described (31–33). WT and Fabry fibroblasts were grown to approximately 70% confluency, and two 150-mm dishes were pooled for each determination. Briefly, fibroblasts were rinsed twice with 8.0 mL of phosphate-buffered saline (PBS, pH 7.4), fixed by adding 0.7 mL of PBS followed by 1.8 mL methanol, and harvested immediately by scraping into a 13 × 100-mm screw-capped glass tube to reduce methanol evaporation. An additional 1.8 mL of methanol was added to collect remaining cellular materials. Total lipids were extracted from cellular materials (approximately 0.5 mL) by adding 1.5 mL of chloroform to yield a theoretical ratio of chloroform:methanol:water in the tube of 1: 2: 0.8 (v/v/v), forming a monophase. After a brief pulse sonication, the sample was centrifuged at 2,400*g* for 30 min to precipitate cellular debris, and the supernatant was transferred to a second screw-capped glass tube (16 × 125 mm). To partition the monophase into an organic phase containing glycosphingolipids and an aqueous phase and to remove unwanted water-soluble lipids, the chloroform:methanol:water ratio in the tube was adjusted to 2:1:0.8 (v/v/v) by adding 4.5 mL of chloroform and 1.2 mL of 0.9% NaCl. After centrifugation at 900*g* for 5 min, the upper aqueous layer was removed, and the lower organic layer containing Gb_3_ and other cellular lipids was washed twice with 3.0 mL of methanol and 2.4 mL of 0.9% NaCl and then dried under a stream of nitrogen gas (N_2_).

After colorimetric determination of total inorganic phosphate (Pi) in lipid extracts, a portion of crude lipids from each sample, normalized to 100 nmol of total phospholipid phosphate, was dried down under N_2_, and the dried lipids were incubated in 2.0 mL of chloroform and 1.0 mL of 0.21 N NaOH in methanol (monophase) at room temperature (RT) for 1 h to subject contaminating glycerophospholipids to alkaline methanolysis in the extract. The alkaline hydrolysis was neutralized by adding 0.8 mL of 0.25 N HCl, yielding organic and aqueous layers. The aqueous layer was discarded after centrifugation at 900*g* for 5 min. The lower 2.0 mL chloroform phase, containing neutral glycosphingolipids, was mixed with 4 mL of methanol and incubated with 1.6 mL of an acid hydrolysis solution consisting of 0.05 N HCl and 25 mM HgCl_2_ at RT for 30 min. The acid hydrolysis was terminated by adding 2.0 mL of chloroform and 1.6 mL of double-distilled water (dd water). The upper aqueous layer was removed after centrifugation at 900 × g for 5 min, and the lower organic layer was purified once with 2.0 mL of methanol and 1.6 mL of 30 mM EDTA and twice with 2.0 mL of methanol and 1.6 mL of dd water. The glycosphingolipids in chloroform solution were evaporated under N_2_ and analyzed by HPTLC. The purified and dried glycosphingolipid residues were resuspended in 50 µL of chloroform:methanol (2:1, v/v), spotted on an HPTLC plate, and separated using a two-solvent system. The plates were first developed by dipping them in 100 mL of chloroform:methanol (98:2, v/v) for 25 min and then developed in 100 mL of chloroform:methanol:water:acetic acid:NH_4_OH (64:31:3:2:0.5, v/v/v/v/v) for 40 min. Glycosphingolipids were visualized by charring with 8% cupric sulfate in methanol:water:H_3_PO_4_ (8:60:32:8, wt./v/v/v). The positions of Gb_3_ were identified by comparison with authentic standards (Matreya, PA) run in parallel. The intensity of Gb_3_ bands was quantified by densitometric scanning using NIH ImageJ 1.62 software. Data were pooled from three independent experiments (*n* = 3).

### Fibroblast culture and reprogramming

Studies using hiPSC lines were conducted with institutional approval from the Human Pluripotent Stem Cell Oversight Committee of the University of Michigan.

The wild-type (WT) human induced pluripotent stem cell (hiPSC) line (iPS-DF19-9-11T.H) was obtained from the WiCell Research Institute. Three dermal fibroblast cell lines were obtained from the Corriell Institute for Medical Research biobank and included the following: GM00881, derived from a white male donor with no detectable α-galactosidase A activity (c.658C>T; R220X); GM02771, derived from a female donor with 50% of α-galactosidase A activity (c.658C>T; R220X); and GM00107, derived from a white male donor with 15% of normal α-galactosidase A activity (c.485G>A; W162X). Fibroblasts were cultured on Matrigel-coated plates in fibroblast maintenance medium (DMEM:F12 supplemented with 10% FBS, 1 mmol/L L-glutamine, 0.1 mmol/L non-essential amino acids, and 0.1 mmol/L β-mercaptoethanol) until reaching 80% confluency for reprogramming. Sendai virus carrying the Yamanaka factors (CytoTune 2.0, Invitrogen) was used to reprogram dermal fibroblasts into hiPSCs. Briefly, cells were exposed to Sendai virus for 24 h and cultured in fibroblast medium, which was replaced every other day. Seven days after transduction, the fibroblast medium was replaced with hiPSC medium (StemMACS iPSC-Brew XF, Miltenyi Biotech). Cells were passaged onto a new Matrigel-coated plate to prevent overconfluence, and the hiPSC medium was changed daily. Emerging hiPSC colonies were isolated individually for clonal expansion and subsequent cryopreservation. New hiPSC lines were verified by immunofluorescence staining for pluripotent stem cell-specific markers, including OCT3/4, SOX2, and NANOG; furthermore, the cells were karyotyped at the WiCell Research Institute. If aneuploidies were detected in the hiPSC lines, the corresponding fibroblast lines were submitted for karyotyping.

### hiPSC maintenance, cardiac differentiation, and cardiomyocyte purification

hiPSCs were maintained, expanded, and differentiated as described previously (34, 35). Briefly, hiPSC colonies were cultured on Matrigel-coated plastic six-well plates using hiPSC medium (StemMACS iPSC-Brew XF, Miltenyi Biotech). Laboratory personnel visually assessed hiPSC plates daily, with manual marking and picking to remove spontaneous differentiation. Plates reaching 70% confluency were passaged using an EDTA solution (Versene, Gibco) for 5 min. Colonies were then resuspended and diluted in hiPSC medium for replating either at a lower density for maintenance or at a higher density to form monolayers for differentiation.

Monolayers were differentiated into hiPSC-derived ventricular cardiomyocytes (hiPSC-VCMs) using the protocol developed by Cyganek et al. (36) and reproduced in our laboratory (34, 35). Briefly, hiPSC monolayers reaching 90%–100% confluence were washed with Hank's Balanced Salt Solution (HBSS) and exposed to basal medium (RPMI supplemented with 25 mM HEPES, 0.5 mg/mL L-ascorbic acid-2-phosphate, and 0.25 mg/mL bovine serum albumin) supplemented with CHIR99021 (4 µM) for 2 days. On day 2, monolayers were switched to basal medium supplemented with IWP4 (4 µM) to induce cardiac mesoderm for 2 days. On days 4 and 6, monolayers were fed with basal medium without supplements, and on day 8, after initiation of differentiation, cells were switched to cardiomyocyte maintenance medium (RPMI supplemented with complete B27).

Cells were observed for beating, and purification was performed using magnetic-assisted cell sorting (MACS) as previously described (34, 35, 37, 38). Differentiated cells were dissociated with 0.25% trypsin/EDTA for 10 min and suspended in plating medium (DMEM:F12 supplemented with 10% FBS, 1 mmol/L L-glutamine, 0.1 mmol/L non-essential amino acids, 0.1 mmol/L β-mercaptoethanol, and 25 µM blebbistatin) to inactivate trypsin. Suspended cells were centrifuged and washed with MACS separation buffer (Miltenyi Biotech) before incubation with a primary antibody cocktail designed for the negative selection of cardiomyocytes (PSC-derived cardiomyocyte isolation kit, Miltenyi). After incubation with primary antibodies, cells were washed with MACS separation buffer and incubated with secondary antibodies conjugated to magnetic beads. The cell suspension was applied to magnetic columns, and the flow-through containing cardiomyocytes was collected. Cells were counted, pelleted, and resuspended in plating medium to provide a plating cell density of 7.5 × 10^4^ cells/well in a 96-well plate. hiPSC-VCM maturation was induced by plating monolayers on human extracellular matrix-coated 96-well plates (MatrixPlus, StemBioSys, San Antonio, TX). hiPSC-CMs were cultured for 48 h in plating medium before switching to cardiomyocyte maintenance medium. Media changes were performed every other day until 7 days after purification. A step-by-step video of the cardiac differentiation procedure, cardiomyocyte purification, plating, and maintenance of hiPSC-VCMs is available in one of our prior publications (34).

### High-throughput cardiac electrophysiology optical mapping

High-throughput optical mapping to detect changes in membrane voltage or intracellular calcium was performed as previously described (34, 35, 37–40). Functional syncytia of mature cardiomyocytes were loaded with Fluovolt (Catalog# F10488; Thermo Fisher) for voltage mapping by dissolving Fluovolt in PowerLoad (1:10) and diluting the Fluovolt/PowerLoad mixture in HBSS (1:100). Cells were incubated with the dye for 30 min at 37°C and washed with HBSS before acquisition of optical mapping movies. CalBryte 520AM (5 µM, ATTC, USA) was used for intracellular calcium mapping. Briefly, Calbryte 520AM was diluted in HBSS, applied to the functional syncytia for 30 min at 37°C, and washed with HBSS before acquisition of electrophysiological data.

Cardiac electrophysiology data were acquired as previously described (Cartox, 100 fps, 10–20 s movies) (35). Briefly, fluorescence readings were obtained by placing multi-well plates on a temperature-controlled motorized XY stage maintained at 37 °C ± 0.5 °C. Excitation illumination was provided by a high-power LED array (470 nm; excitation filter ET470/40x), and fluorescence signals were recorded from a 3.5 × 2.0-cm area using a high-numerical-aperture camera lens fitted with a band-pass emission filter (ET525/50M) positioned in front of a high-speed sCMOS camera sensor (DaVinci2K, SciMeasure) (35). Optical mapping movies were analyzed using commercial software (StemBioSys Optical Electrophysiology Tool) and stored as .oeat files. Main parameters calculated with this software included the frequency of spontaneous depolarization, action potential duration at 80% repolarization (APD_80_), APD triangulation (APD_tri_ = APD_90_−APD_30_), AP upstroke slope, and conduction velocity of spontaneous activations (Supplementary Figure S1A). Similarly, intracellular calcium mapping data were analyzed to calculate the frequency of spontaneous activations, intracellular calcium transient duration at 80% return to baseline (CaTD_80_), CaTD triangulation (CaTD_tri_ = CaTD_90_−CaTD_30_), maximal rate of calcium rise, conduction velocity, baseline (diastolic calcium) fluorescence, and calcium transient amplitude (Supplementary Figure S1B).

### Adenovirus-mediated overexpression of α-galactosidase A

For cellular expression of α-Gal A, a commercially available adenovirus carrying *GLA* gene under the control of a CMV promoter (Vector Biolabs, USA) was used. The optimal multiplicity of infection (MOI) was confirmed by testing different MOIs in a 96-well plate of FD hiPSC-VCMs. The efficacy of each MOI was assessed by immunofluorescence staining for Gb_3_ 7 days after adenoviral transduction of mature hiPSC-VCM monolayers. Briefly, cells fixed with 2% paraformaldehyde were permeabilized (PBS supplemented with 0.3% Triton X-100) and blocked with 10% normal goat serum in PBS. hiPSC-VCMs were incubated with anti-Gb3 antibody (1:100; A2506, TCI, USA) in PBS supplemented with 5% goat serum overnight at 4˚C. After removal of the primary antibody, hiPSC-VCMs were incubated with Alexa Fluor 594-conjugated goat anti-mouse IgG antibody for 1 h at room temperature and counterstained with DAPI. The optimal MOI was then applied to FD hiPSC-VCMs and WT hiPSC-VCMs, and these treatment groups were designated as adv-GLA FD hiPSC-VCMs and adv-GLA WT hiPSC-VCMs, respectively.

### mRNA extraction, mRNA sequencing, and bioinformatics analysis

FD hiPSC-VCMs, WT hiPSC-VCMs, adv-GLA FD hiPSC-VCMs, and adv-GLA WT hiPSC-VCMs were cultured on MatrixPlus-coated six-well plates (1.2 × 10^6^ cells/well) for 7 days after adenoviral transduction. hiPSC-CM monolayers were collected in QIAzol, purified using RNeasy columns, and quantified using a NanoDrop spectrophotometer prior to submission to the University of Michigan Advanced Genomics Core. The Advanced Genomics Core performed poly(A) enrichment using the NEB Poly(A) kit to enrich for mRNA prior to cDNA synthesis and library preparation with the IDT xGen Broad-Range RNA Library Prep Kit. Sequencing was performed on a NovaSeq at PE150, targeting 30–40 million reads per sample. This approach provides information on expression and splicing/isoform. Data were delivered as demultiplexed FASTQ files, aligned read BAM files, preliminary QC reports, and a table of gene read counts (suitable for differential expression analysis) for use in the bioinformatic pipeline for analysis at the University of Michigan Bioinformatics Core.

Snakemake (41) was used to manage the bioinformatics workflow in a reproducible manner at the Bioinformatics Core of the University of Michigan. Data were pre-filtered to remove genes with 0 counts across all samples. Differential gene expression analysis was performed using DESeq2 (42) with a negative binomial generalized linear model [thresholds: linear fold change >1.5 or ≤1.5, Benjamini–Hochberg FDR (*P*_adj_) < 0.05]. Plots were generated using variations of the DESeq2 plotting functions and other packages in R (version 4.1.3). ENSEMBL 105 and Entrez Gene IDs were used for gene annotation. Functional analysis, including identification of candidate pathways that were activated or inhibited in the comparison(s) and GO term enrichments (43), was performed using iPathwayGuide (44).

### Statistical analysis

Statistical analyses and graphing were performed using GraphPad. The distribution of quantitative data was investigated using the Kolmogorov–Smirnov test. Homoscedasticity of the data was evaluated using the *F*-test for variances. Multiple comparisons of quantitative data confirming the statistical premises were performed using ANOVA followed by Tukey's test for means. Significance levels were adjusted for multiple testing using Bonferroni's correction. Multiple comparisons of quantitative data that did not conform to statistical premises were carried out using the Kruskal–Wallis test, followed by the Mann–Whitney *U*-test for group-to-group comparisons. Significance was adjusted using Bonferroni's correction. Associations between qualitative variables were determined using the chi-square test. Data are presented as mean ± standard error of the mean.

## Results

**Figure 1:**
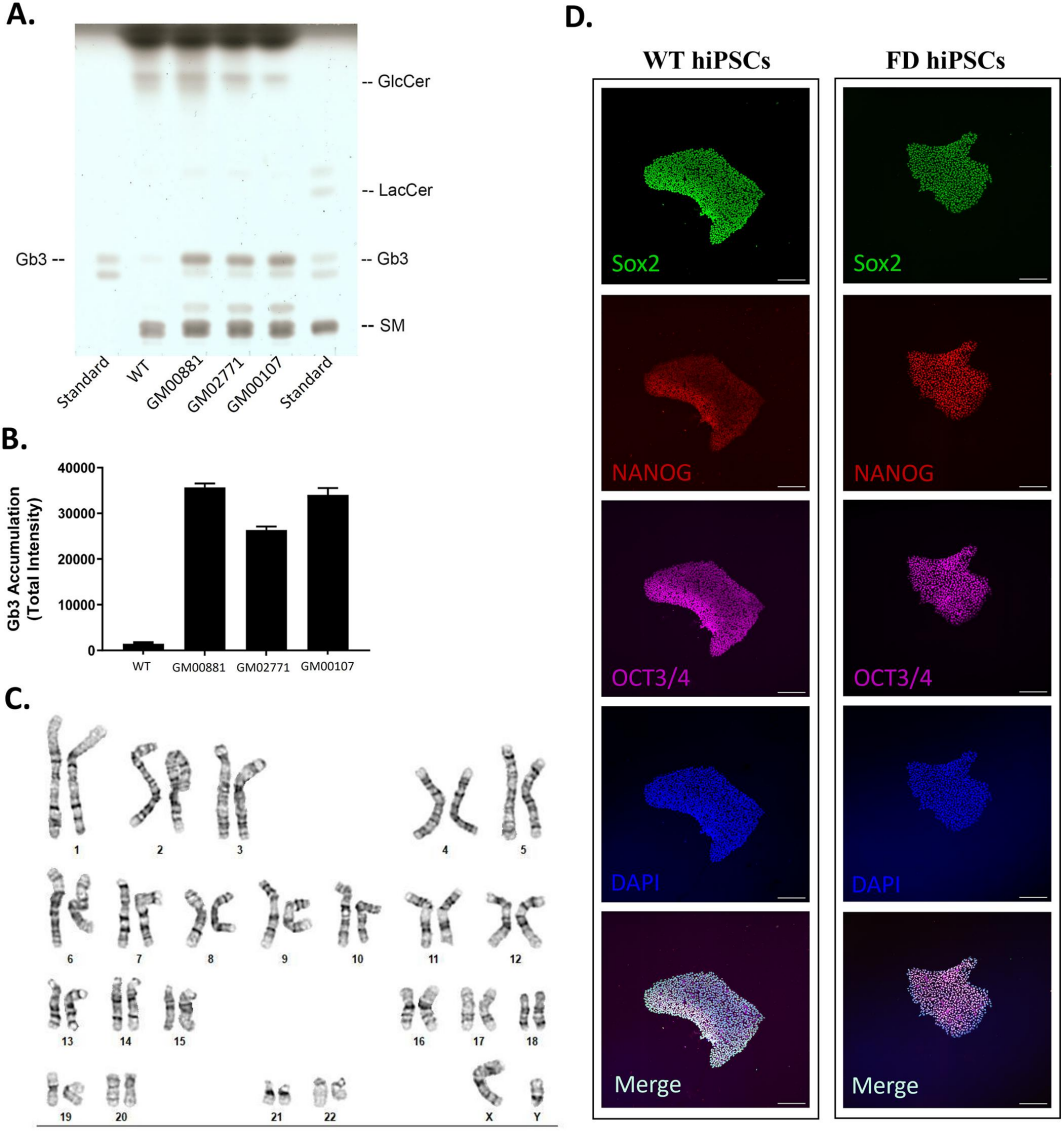
hiPSCs derived from a Fabry disease patient. **(A,B)** Thin-layer chromatography of wild-type and Fabry disease (FD) fibroblasts demonstrating Gb_3_ accumulation in FD fibroblasts (GM00881, GM02771, and GM00107). **(C)** Normal karyotype of FD hiPSCs generated from GM00881 fibroblasts. **(D)** WT and FD hiPSCs showing comparable expression of pluripotency markers (SOX2, NANOG, and OCT3/4).

Gb_3_ accumulation, a hallmark of FD, was measured in patient-derived dermal fibroblasts. High-performance thin-layer chromatography indicated that dermal fibroblasts from FD patients exhibited significantly higher accumulation of Gb_3_ compared with wild-type (WT) fibroblasts (Figures 1A,B), consistent with decreased GLA activity. Following confirmation of Gb_3_ accumulation, dermal fibroblasts from Fabry disease patients were reprogrammed into human induced pluripotent stem cells (hiPSCs) using a Sendai virus carrying the Yamanaka factors.

Reprogramming of fibroblasts into human induced pluripotent stem cells (Supplementary Movie S1) was successful in all three cell lines from Fabry Disease patients (GM00881, GM02771, and GM00107; Coriell Institute for Medical Research); however, during karyotype analysis, two of the newly reprogrammed cell lines (GM02771 and GM00107) exhibited aneuploidies. To determine whether these aneuploidies were artifacts of the reprogramming process, we submitted dermal fibroblasts from GM02771 and GM00107 for karyotyping and confirmed that the chromosomal aberrations were already present in the dermal fibroblasts prior to Sendai virus-mediated reprogramming. The euploid Fabry disease hiPSC line (GM00881, FD; Figure 1C) showed appropriate expression of pluripotency markers (OCT3/4, NANOG, and SOX2; Figure 1D). Furthermore, these cells were successfully differentiated into hiPSC-derived cardiomyocytes using our current ventricular cardiomyocyte differentiation protocol (34, 35).

**Figure 2:**
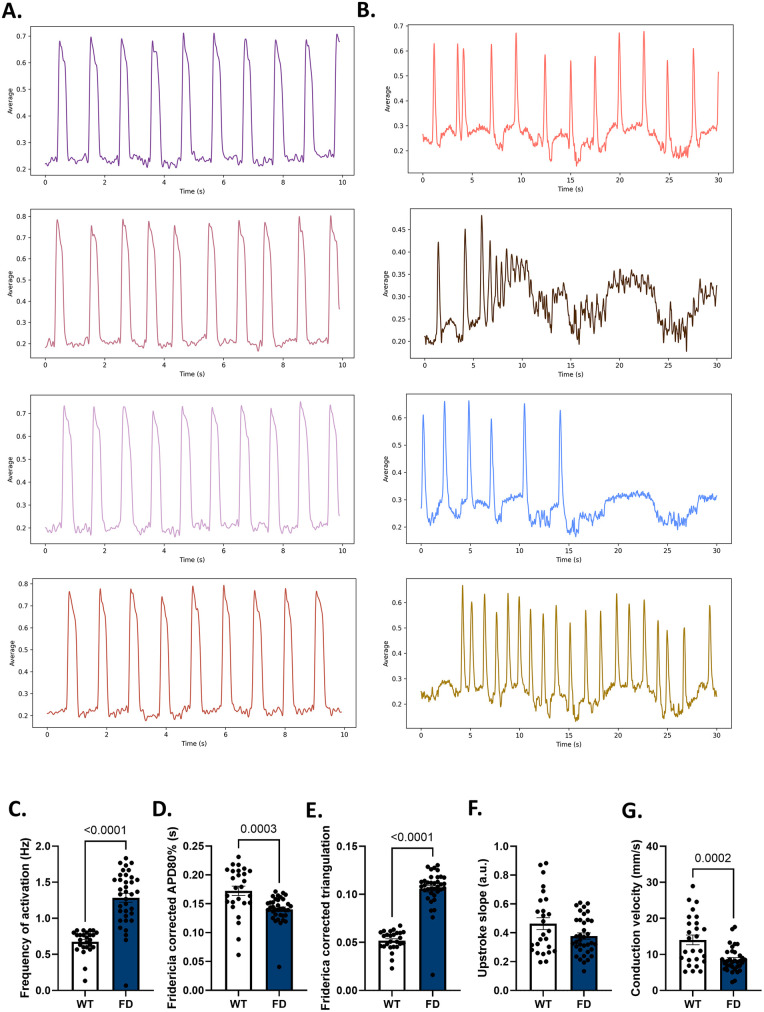
Assessment of action potentials in WT and FD hiPSC-VCMs. **(A)** Representative traces showing normal spontaneous activation in WT hiPSC-VCMs. **(B)** Representative traces showing chaotic arrhythmic activity in FD hiPSC-VCMs. **(C)** Frequency of spontaneous activations was significantly higher in FD hiPSC-VCMs than in WT-hiPSC-VCMs. (D) The Fridericia-corrected APD_80_ was longer in WT hiPSC-VCMs than in FD hiPSC-VCMs. **(E)** APD triangulation was longer in FD hiPSC-VCMs than in WT hiPSC-VCMs. **(F)** Upstroke slope of the action potential was similar between WT and FD hiPSC-VCMs. **(G)** Conduction velocity of action potential propagation was significantly slower in FD hiPSC-VCMs.

Ventricular hiPSC-derived cardiomyocytes (hiPSC-VCMs), purified using magnetic-assisted cell sorting to avoid metabolic damage (37), were replated on maturation-inducing extracellular matrix to form mature functional syncytia (34, 35, 39) before optical mapping for detection of action potential. Optical mapping of WT hiPSC-VCMs did not reveal arrhythmias (0/36 functional syncytia; Figure 2A); in contrast, arrhythmias were present in all matured functional syncytia derived from Fabry disease hiPSC-VCMs (FD hiPSC-VCMs, 16/16 functional syncytia; Figure 2B), including re-entrant arrhythmias (Supplementary Movie S2). Due to prolonged periods of fibrillation or arrest, optical mapping of FD hiPSC-CMs had to be performed for 30 s, whereas WT hiPSC-VCMs required only 10 s of analysis.

Further studies involving an increased number of functional syncytia of WT and FD hiPSC-VCMs subjected to optical mapping for detection of voltage changes indicated that the frequency of spontaneous activation was higher in FD hiPSC-VCMs (1.28 ± 0.06 Hz; *n* = 26) than in WT hiPSC-VCMs (0.67 ± 0.03 Hz, *n* = 36; *p* < 0.0001, Figure 2C). Because of this difference in the frequency of spontaneous activation, we performed a Fridericia correction of the action potential duration. Action potential duration at 80% repolarization (APD_80_) was longer in WT hiPSC-VCMs (0.17 ± 0.008s) than in FD hiPSC-VCMs (0.14 ± 0.003 s, *p* = 0.0003; Figure 2C). However, assessment of phase 3 repolarization with Fridericia-corrected APD triangulation (APD_tri_ = APD_90_ − APFD_30_) indicated that FD hiPSC-VCMs had a greater APDtri (FD = 0.10 ± 0.003 s) compared with WT hiPSC-VCMs (0.05 ± 0.002 s, *p* < 0.0001; Figure 2D). The upstroke slope of the action potential was similar between the two groups (WT hiPSC-VCMs = 0.46 ± 0.04 a.u. and FD hiPSC-VCMs = 0.377 ± 0.02 a.u., *p* = 0.051; Figure 2E); however, there was a significant reduction in conduction velocity of the action potential in FD hiPSC-VCMs (8.5 ± 0.5 cm/s) compared with WT hiPSC-VCMs (14 ± 1.3 cm/s, *p* = 0.0002; Figure 2F).

**Figure 3:**
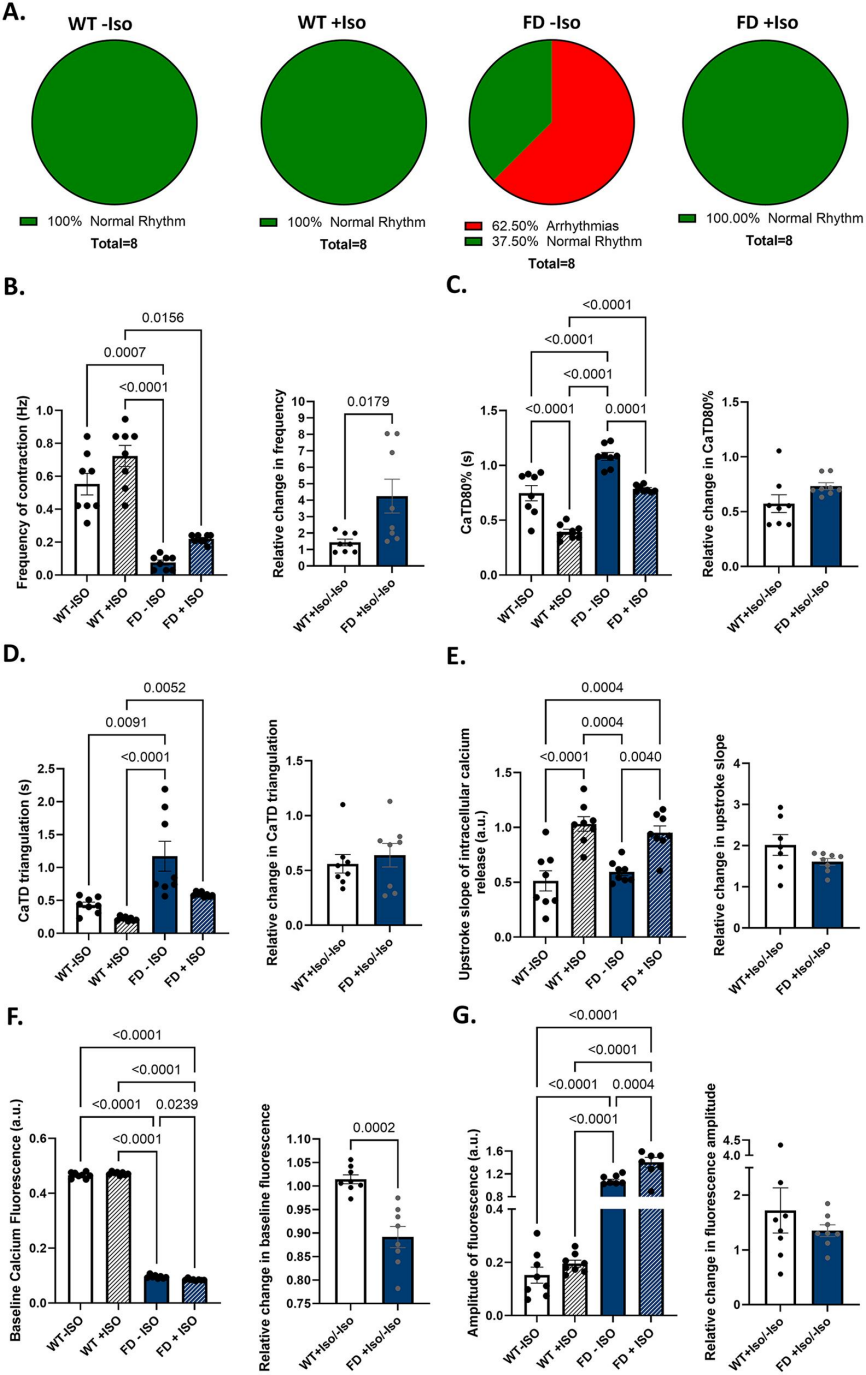
Response of hiPSC-VCMs to β-adrenergic stimulation. **(A)** Functional syncytia of WT hiPSC-VCMs did not exhibit arrhythmias before and after isoproterenol (Iso, 100 nM) treatment. In contrast, FD hiPSC-VCMs showed a high incidence of arrhythmias before isoproterenol treatment, which were absent after treatment. **(B)** Although the frequency of intracellular calcium transients was lower in FD hiPSC-VCMs than in WT hiPSC-VCMs before and after isoproterenol (100 nM) treatment; however, after treatment, the relative change (post-iso/pre-iso) in contraction frequency was significantly greater in FD compared with WT hiPSC-VCMs. **(C)** CaTD_80_ was significantly longer in FD hiPSC-VCMs than in WT hiPSC-CM before and after isoproterenol treatment; however, the relative changes were similar between groups. **(D)** Calcium transient triangulation duration (CaTD_tri_ = CATD_90_-CaTD_30_) was shorter in WT hiPSC-VCMs than in FD hiPSC-VCMs before and after isoproterenol treatment; however, the relative change in CaTD_tri_ was similar between groups. **(E)** Upstroke of intracellular calcium release was similar between WT hiPSC-VCMs and FD hiPSC-VCMs before and after isoproterenol treatment, and the relative change in the upstroke slope was similar between groups. **(F)** Baseline calcium fluorescence was higher in WT hiPSC-VCMS than in FD hiPSC-VCMs before and after isoproterenol treatment; however, the relative change in baseline fluorescence was similar between groups. **(G)** Amplitude of calcium fluorescence was higher in FD hiPSC-VCMs than in FD hiPSC-VCM before and after isoproterenol treatment; however, the relative change in calcium fluorescence amplitude was similar between groups.

To investigate the response of hiPSC-VCMs to flight-or-fight stress, β-adrenergic stimulation was performed using isoproterenol treatment (Iso, 100 nM). Wild-type hiPSC-VCMs did not exhibit arrhythmias before Iso treatment; however, 62.5% of the functional syncytia derived from FD hiPSC-CMs displayed arrhythmias (Figure 3A). Importantly, treatment with Iso established a rhythmic pattern of activation in FD hiPSC-VCMs syncytia (Figure 3A). The frequency of contraction (measured as intracellular calcium transients) was lower in FD hiPSC-VCMs before (0.07 ± 0.014 Hz; *n* = 8) and after treatment with 100nM Iso (0.21 ± 0.009 Hz; *n* = 8) compared with WT hiPSC-VCMs before (0.55 ± 0.065 Hz, *n* = 8) and after treatment (0.72 ± 0.064 Hz, *n* = 8; Figure 3B). However, the rate of change (post-iso/pre-iso) in contraction frequency was significantly higher in FD hiPSC-VCMs (4.2 ± 1, *n* = 8) than in WT hiPSC-VCMs (1.45 ± 0.2, *n* = 8, *p* = 0.017; Figure 3B).

Calcium transient duration at 80% return to baseline (CaTD_80_) was shortened by Iso treatment in both WT hiPSC-VCMs (WT − Iso = 0.74 ± 0.069 s, WT + Iso = 0.39 ± 0.024 s; *p* < 0.0001) and FD hiPSC-VCMs (FD − Iso = 1.08 ± 0.035 s, FD + Iso = 0.78 ± 0.01 s; *p* = 0.0001; Figure 3C). Despite the significant differences in CaTD_80_ between WT and FD hiPSC-VCMs, the relative change in CaTD_80_ following Iso treatment was similar between the groups (*p* = 0.06; Figure 3C). The duration of intracellular calcium reuptake into the sarcoplasmic reticulum was assessed using CaT triangulation (CaTD_tri_ = CATD_90_−CaTD_30_). Before Iso treatment, WT hiPSC-VCMs exhibited a shorter CaTD_tri_ (0.43 ± 0.042 s) compared with FD hiPSC-VCMs (1.172 ± 0.22 s, *p* = 0.009). After Iso treatment, CaTD_tri_ remained shorter in WT hiPSC-VCMs (0.22 ± 0.01 s) than in FD hiPSC-VCMs (0.58 ± 0.012 s, *p* = 0.005; Figure 3D). However, the rate of change in CaTD_tri_ was similar between WT hiPSC-VCMs (0.56 ± 0.08 s) and FD hiPSC-VCMs (0.64 ± 0.1, *p* = 0.5; Figure 3D). The rate of intracellular calcium rise was assessed using the upstroke slope of the intracellular calcium transient. The upstroke of intracellular calcium release was similar between WT hiPSC-VCMs (0.51 ± 0.09 a.u.) and FD hiPSC-VCMs (0.59 ± 0.03 a.u., *p* = 0.8; Figure 3E) before and after Iso treatment (WT + Iso = 1.03 ± 0.06 and FD + Iso=0.95 ± 0.06 a.u, *p* = 0.8; Figure 3E).

Baseline calcium fluorescence was used as a surrogate for diastolic calcium levels. Before Iso treatment, baseline calcium fluorescence was higher in WT hiPSC-VCMs (0.46 ± 0.003) than in FD hiPSC-VCMs (0.09 ± 0.002, *p* < 0.0001; Figure 3F), and after Iso treatment, baseline calcium fluorescence remained higher in WT hiPSC-VCMs (0.47 ± 0.001) than in FD hiPSC-VCMs (0.08 ± 0.001, *p* < 0.0001; Figure 3F). However, FD hiPSC-VCMs showed a decrease in the relative change in baseline calcium fluorescence (0.89 + 0.02) compared with WT hiPSC-VCMs (1.01 + 0.009, *p* = 0.0002; Figure 3F). The Iso-induced change in the calcium fluorescence amplitude was similar between WT hiPSC-VCMs (1.72 ± 0.4) and FD hiPSC-VCMs (1.35 ± 0.1, *p* = 0.4; Figure 3G), despite differences in calcium transient amplitude between WT and FD hiPSC-VCMs before Iso treatment (WT − Iso = 0.15 ± 0.03 vs. FD − Iso = 1.05 ± 0.04, *p* < 0.0001; Figure 3G) and after Iso treatment (WT + Iso = 0.19 ± 0.01 vs. FD + Iso = 1.4 ± 08, *p* < 0.0001; Figure 3G).

**Figure 4:**
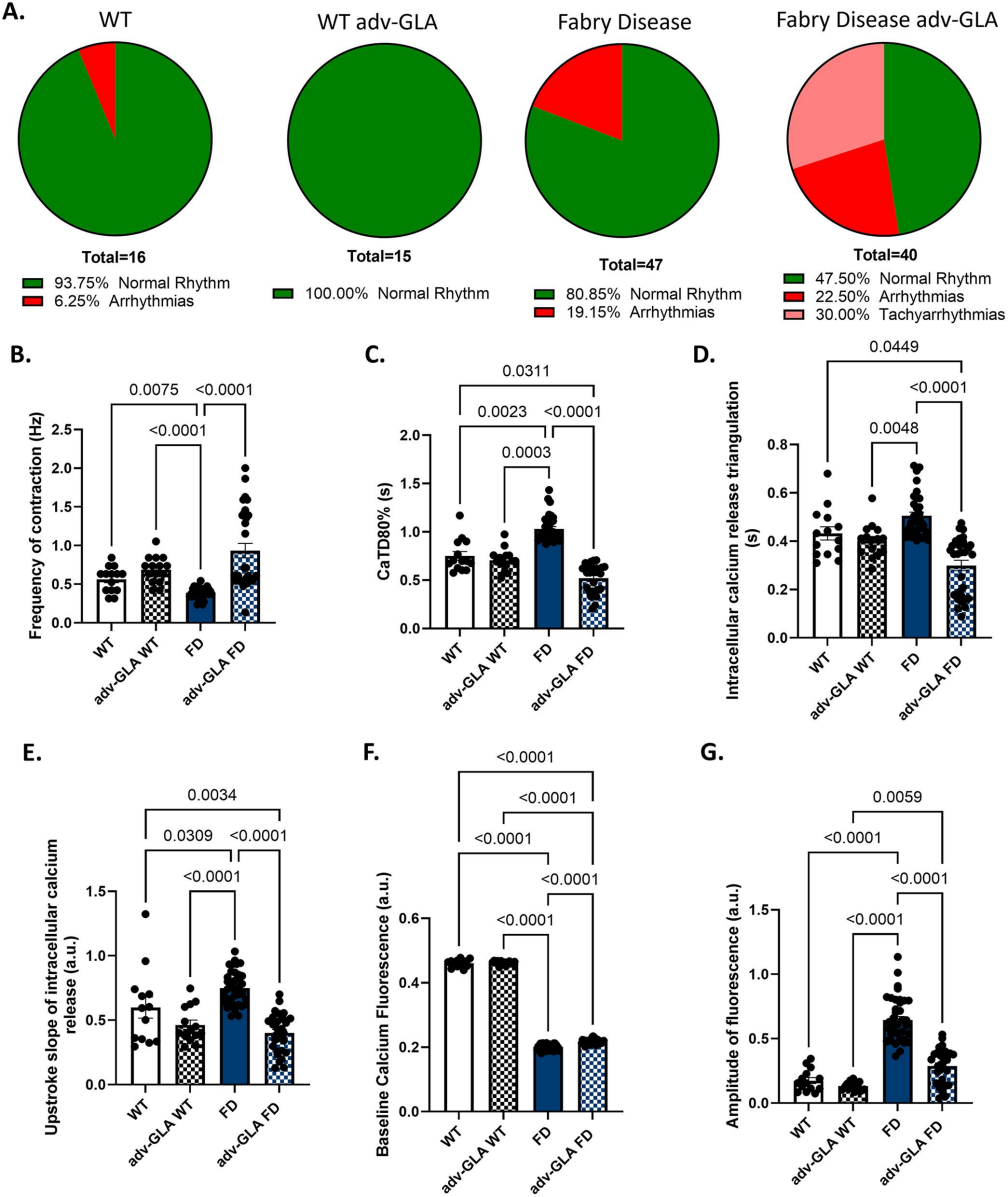
Effects of GLA overexpression on arrhythmias. **(A)** Incidence of spontaneous arrhythmias was similar between naïve and adv-GLA-treated WT hiPSC-VCMs; however, adv-GLA treatment did not reduce the high incidence of spontaneous arrhythmia in FD hiPSC-VCMs and induced tachyarrhythmias, characterized by an abnormal increase in the frequency of spontaneous activation. **(B)** Frequency of spontaneous contractions was lower in FD hiPSC-VCM than in both groups of WT hiPSC-VCMs, and treatment with adv-GLA significantly increased the frequency of spontaneous activation. **(C)** Treatment with adv-GLA did not affect CaTD_80_ in WT hiPSC-VCMs. FD hiPSC-VCMs exhibited prolonged CaTD_80_ compared with WT hiPSC-VCMs, and treatment with adv-GLA significantly reduced CaTD_80_ relative to all groups. **(D)** CaTD_tri_ of WT hiPSC-VCMs was not affected by adv-GLA treatment. CaTD_tri_ was similar between WT and FD hiPSC-VCMs; however, CaTD_tri_ was shorter in adv-GLA WT than in FD hiPSC-VCMs. Adenovirus-mediated overexpression of GLA shortened CaTD_tri_ in adv-GLA FD hiPSC-VCMs compared with WT and FD hiPSC-VCMs but not compared with adv-GLA WT hiPSC-VCMs. **(E)** Upstroke of intracellular calcium release was similar between WT and adv-GLA hiPSC-VCMs, but it was higher in FD hiPSC-VCMs; treatment with adv-GLA significantly reduced the upstroke slope in FD hiPSC-VCMs. **(F)** Treatment with adv-GLA did not alter baseline calcium fluorescence in WT hiPSC-VCMs. FD hiPSC-VCMs exhibited significantly lower baseline calcium fluorescence, which increased slightly after adv-GLA treatment. **(G)** Amplitude of intracellular calcium fluorescence was not impacted by adv-GLA treatment in WT hiPSC-VCMs. FD hiPSC-VCMs showed a significantly higher amplitude of calcium fluorescence compared with WT hiPSC-VCMs. adv-GLA WT and adv-GLA FD hiPSC-VCMs. Adv-GLA WT, wild type hiPSC-VCMs treated with adenovirus for overexpression of GLA; Adv-GLA FD, Fabry Disease hiPSC-VCMs treated with adenovirus for overexpression of GLA.

Next, adenovirus-mediated overexpression of GLA (adv-GLA) was performed to treat FD hiPSC-CMs. We applied 10 MOI of adv-GLA, which induced a 10 log_2_FC in GLA expression in the treated groups, and assessed the effect of GLA rescue on arrhythmias and intracellular calcium handling. In this experiment, overexpression of GLA in WT hiPSC-VCMs did not alter the incidence of arrhythmias relative to WT hiPSC-VCMs (WT = 0%; 0/15 vs. GLA-WT = 6.25%; 1/16; *p* = 0.99). The incidence of arrhythmias (early and delayed premature calcium release, or sudden arrest; Supplementary Figure S2) in FD hiPSC-VCMs (19.15%, 9/47) was similar to that observed in adv-GLA FD hiPSC-VCMs (22.5%, 9/40; *p* = 0.7; Figure 4A). Furthermore, the adv-GLA FD hiPSC-VCM group displayed a significantly higher number of functional syncytia with tachyarrhythmias (30%; 12/40, Figure 4A).

The spontaneous rate of contraction in FD hiPSC-VCMs (0.38 ± 0.01 Hz) was lower than in WT hiPSC-VCMs (0.56 ± 0.04 Hz; *p* = 0.0075) and adv-GLA WT hiPSC-VCMs (0.68 ± 0.04 Hz, *p* = 0.0001); however, adv-GLA treatment significantly increased the spontaneous rate of contraction in FD hiPSC-VCMs compared with untreated FD hiPSC-VCMs (*p* < 0.0001; Figure 4B), even when syncytia exhibiting tachyarrhythmias were excluded from the analysis [adv-GLA FD hiPSC-CMs (−tachyarrhythmias) = 0.57 ± 0.01; *p* < 0.0001; see Supplementary Figure S3 for comparison of all parameters]. Duration of calcium transient at 80% return to baseline was not affected by adv-GLA treatment in WT cardiomyocytes (WT = 0.75 ± 0.04 s vs. adv-GLA WT = 0.7 ± 0.02 s; *p* = 0.99). FD hiPSC-VCMs (1.03 ± 0.02 s) had a longer CaTD_80_ than WT hiPSC-VCMs (*p* = 0.002), adv-GLA WT hiPSC-VCMs (*p* = 0.0003), and adv-GLA FD hiPSC-VCMs (0.51 ± 0.02 s, *p* < 0.0001; Figure 4C). Similarly, CaTD_tri_ in WT hiPSC-VCMs was not affected by adv-GLA treatment (WT = 0.43 ± 0.02 s vs. adv-GLA WT = 0.4 ± 0.01 s; *p* = 0.99; Figure 4D). CaTD_tri_ was also similar between WT and FD hiPSC-VCMs (0.5 ± 0.1 s; *p* = 0.07); however, CaTD_tri_ was shorter in adv-GLA WT hiPSC-VCMs than in FD hiPSC-VCMs (*p* = 0.0048). Adenovirus-mediated overexpression of GLA shortened CaTD_tri_ in adv-GLA FD hiPSC-VCMs (0.29 ± 0.02 s) relative to WT hiPSC-VCMs (*p* = 0.04) and FD hiPSC-VCMs (*p* < 0.0001) but not adv-GLA WT hiPSC-VCMs (*p* = 0.29; Figure 4D).

The upstroke slope of intracellular calcium release in WT hiPSC-VCMs was not affected by adv-GLA overexpression (WT 0.59 ± 0.08 a.u. vs. adv-GLA WT 0.46 ± 0.03 a.u., *p* = 0.16; Figure 4E). The upstroke slope in FD hiPSC-VCMs (0.74 ± 0.02) was greater than in WT hiPSC-VCMs (*p* = 0.03), adv-GLA WT hiPSC-VCMs (*p* < 0.0001), and adv-GLA FD hiPSC-VCMs (0.4 ± 0.02, *p* < 0.0001; Figure 4E). Similarly, baseline calcium fluorescence, a surrogate for diastolic calcium, in WT hiPSC-VCMs was not affected by adv-GLA overexpression (WT = 0.461 ± 0.003 a.u. vs. adv-GLA WT = 0.462 ± 0.001 s *p* = 0.97; Figure 4F). Baseline calcium fluorescence was greater in WT hiPSC-VCMs (*p* < 0.0001) and adv-GLA WT hiPSC-VCMs (*p* < 0.0001) compared with FD hiPSC-VCMs (0.201 ± 0.001 a.u.; Figure 4F). Treatment with adv-GLA increased baseline calcium fluorescence in adv-GLA FD hiPSC-VCMs (0.216 ± 0.001; *p* < 0.0001; Figure 4F). The amplitude of the intracellular calcium transient was not affected by adv-GLA treatment in WT hiPSC-VCMs (WT = 0.17 ± 0.02 a.u. vs. adv GLA WT = 0.13 ± 0.009 a.u., *p* = 0.92; Figure 4G); both values were significantly lower than those observed in FD hiPSC-VCMs (0.64 ± 0.02; WT vs. FD, *p* < 0.0001; adv-GLA WT vs. FD, *p* < 0.0001; Figure 4G). Treatment with adv-GLA reduced the amplitude of fluorescence in adv-GLA FD hiPSC-VCMs (0.28 ± 0.024 a.u.) compared with FD hiPSC-CMs (*p* < 0.0001), bringing it closer to the amplitude observed in WT hiPSC-CMs (*p* = 0.073); however, it still remained higher than that observed in adv-GLA WT hiPSC-VCMs (*p* = 0.0059; Figure 4G).

To further understand the impact of GLA expression rescue through adv-GLA transduction of FD hiPSC-VCMs, we performed poly(A)-enriched mRNA sequencing. A total of 3,010 genes were differentially expressed between FD and WT hiPSC-VCMs. Gene Ontology (GO) analysis indicated that the main clusters of molecular function genes affected in FD compared with WT were related to signaling receptor binding (GO:0005102), signaling receptor activity (GO:0038023), molecular transducer activity (GO:0060089), extracellular matrix structural constituent activity (GO:0005201), transmembrane signaling receptor activity (GO:0004888), integrin biding (GO:0005178), calcium ion binding (GO:0005509), signaling receptor activator activity (GO:0030546), receptor ligand activity (GO:0048018), and glycosaminoglycan binding (GO:0005539; Figure 5A).

**Figure 5:**
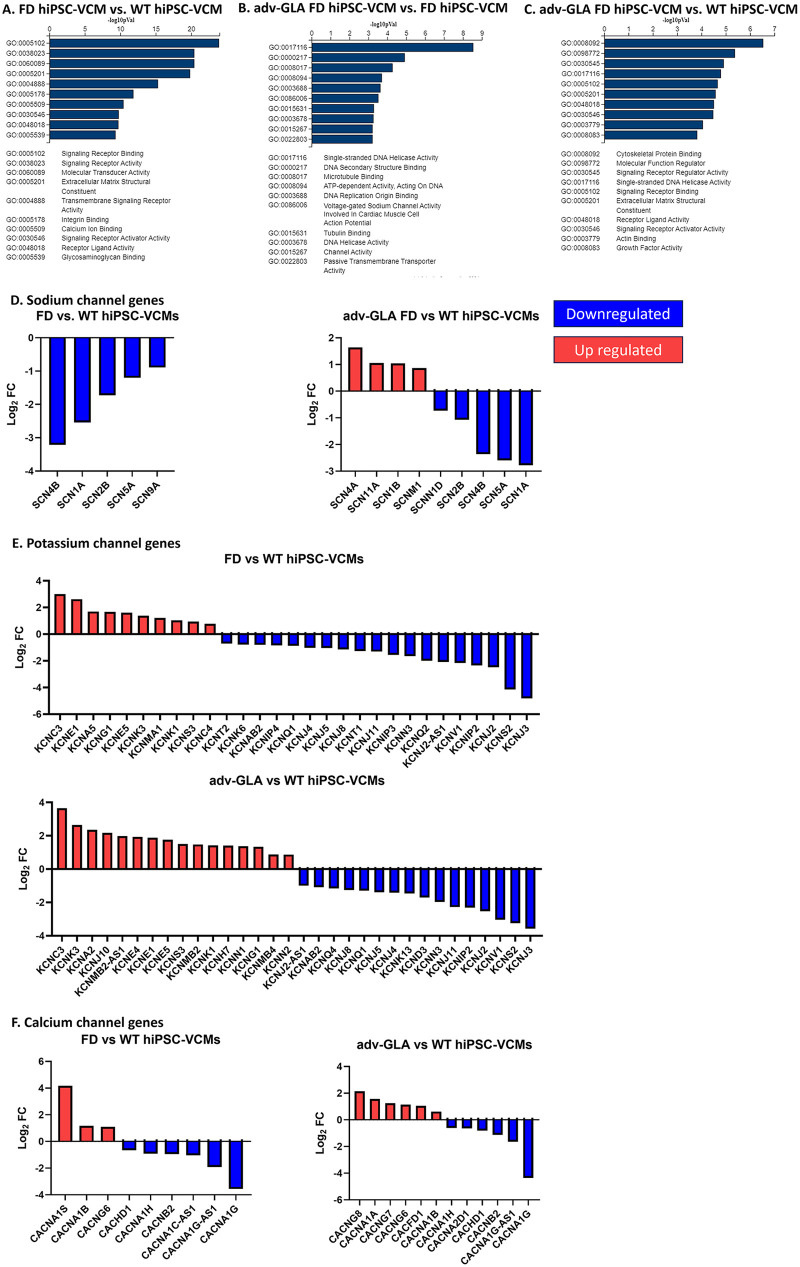
Transcriptomic analysis of the effects of adv-GLA on hiPSC-VCMs. **(A–C)** Gene Ontology molecular function enrichment analysis comparing FD vs. WT, adv-GLA FD vs. FD, and adv-GLA FD vs. WT hiPSC-VCMs. **(D)** Transcripts of several genes encoding sodium channel subunits were differentially expressed between FD and WT hiPSC-CMs; however, transcription of these genes was not equated to WT hiPSC-VCM transcription levels by adv-GLA treatment of FD hiPSC-VCMs. **(E)** Twenty-nine genes encoding potassium channel subunits were differentially regulated between FD and WT hiPSC-VCMs; however, adv-GLA treatment of FD hiPSC-VCMs increased the number of differentially expressed genes to 33 compared with WT hiPSC-VCMs. **(F)** Number of differentially expressed genes encoding calcium channel subunits or anti-sense transcripts between FD and WT hiPSC-VCMs increased from 9 to 12 after adv-GLA treatment of FD hiPSC-VCMs.

Rescue of GLA expression through adv-GLA induced a 10-fold increase (log_2_ fold change) in GLA expression in adv-GLA FD hiPSC-VCMs compared with FD hiPSC-VCMs. Furthermore, a comparison between adv-GLA FD and FD hiPSC-VCMs identified 3,471 differentially expressed genes. Rescue of GLA expression induced qualitative changes in the Gene Ontology molecular function of adv-GLA FD hiPSC-VCMs relative to FD hiPSC-VCMs. Specifically, adv-GLA rescue impacted single-stranded DNA helicase activity (GO:0017116), DNA secondary structure binding (GO:0000217), microtubule binding (GO:0008017), ATP-dependent activity acting on DNA (GO:0008094), DNA replication origin binding (GO:0003688), voltage-gated sodium channel activity involved in cardiac muscle action potential (GO:0086006), tubulin binding (GO:0015631), DNA helicase activity (GO:0003678), channel activity (GO:0015267), and passive transmembrane transporter activity (GO:0022803; Figure 5B). Compared with WT hiPSC-VCMs, adv-GLA WT hiPSC-VCMs exhibited 6,091 differentially expressed genes. The molecular functions affected were cytoskeletal protein binding (GO:0008092), molecular function regulator (GO:0098772), signaling receptor regulator activity (GO:0030545), single-stranded DNA helicase activity (GO:0017116), signaling receptor binding (GO:0005102), extracellular matrix structural constituent activity (GO:0005201), receptor ligand activity (GO:0030546), actin binding (GO:0030546), and growth factor activity (GO:0008083; Figure 5C).

Due to the pronounced arrhythmogenic phenotype of FD hiPSC-VCMs and the failure of adv-GLA to reduce the incidence of arrhythmias, we further refined the RNA sequence analysis to focus on genes encoding sodium, potassium, and calcium channel subunits. A group of genes encoding sodium channel subunits was downregulated in FD hiPSC-VCMs compared with WT hiPSC-VCMs (*SCN4B*, *SCN1A*, *SCN2B*, *SCN5A*, and *SCN9A*; Figure 5D). Although adv-GLA FD hiPSC-VCMs presented increased expression of some SCN genes (*SCN4A*, *SCN11A*, *SCN1B*, and *SCNM1*) compared with WT hiPSC-VCMs, a group of genes encoding sodium channel subunits remained downregulated (*SCNN1D*, *SCN2B*, *SCN4B*, *SCN5A*, and *SCN1A*; Figure 5D). Transcription factors associated with the regulation of SCN5A expression, such as TBX-5 (45–47) and HAND2, were downregulated in FD hiPSC-VCMs copmared with WT hiPSC-VCMs (HAND2 log_2_FC = −1.17, *p* < 0.0001; TBX5 log_2_FC = −0.75, *p* < 0.0001), and the expression of these genes was not rescued by adv-GLA treatment when compared with WT (HAND2 log_2_FC = −1.81, *p* < 0.0001; TBX5 log_2_FC = −1.03, *p* < 0.0001).

Furthermore, several genes encoding potassium channel subunits were differentially expressed in FD hiPSC-VCMs compared with WT hiPSC-VCMs (10 genes were upregulated: *KCNC3*, *KCNE1*, *KCNA5*, *KCNG1*, *KCNE5*, *KCNK3*, *KCNMA1*, *KCNK1*, *KCNS3*, and *KCNC4*, whereas 19 genes were downregulated: *KCNT2*, *KCNK6*, *KCNAB2*, *KCNIP4*, *KCNQ1*, *KCNJ4*, *KCNJ5*, *KCNJ8*, *KCNT1*, *KCNJ11*, *KCNIP3*, *KCNN3*, *KCNQ2*, *KCNJ2-AS1*, *KCNV1*, *KCNIP2*, *KCNJ2*, *KCNS2*, and *KCNJ3*; Figure 5E). Treatment of FD hiPSC-VCMs with adv-GLA did not reduce the amount of differentially regulated genes encoding potassium channel subunits (16 genes were upregulated: *KCNC3*, *KCNK3*, *KCNA2*, *KCNJ10*, *KCNMB2-AS1*, *KCNE4*, *KCNE1*, *KCNE5*, *KCNS3*, *KCNMB2*, *KCNK1*, *KCNH7*, *KCNN1*, *KCNG1*, *KCNMB4*, and *KCNN2*, whereas 16 genes were downregulated: *KCNJ2-AS1*, *KCNAB2*, *KCNQ4*, *KCNJ8*, *KCNQ1*, *KCNJ5*, *KCNJ4*, *KCND3*, *KCNK13*, *KCNN3*, *KCNJ11*, *KCNIP2*, *KCNJ2*, *KCNV1*, *KCNS2*, and *KCNJ3*; Figure 5E). Transcription factors regulating the expression of genes encoding potassium channel subunits were disrupted in FD hiPSC-VCMs compared with WT hiPSC-VCMs (ETS-1 log_2_FC = 2.38; TBX5 log_2_FC = −0.75, *p* < 0.0001), and rescue of FD hiPSC-VCMs with adv-GLA did not alter the differential expression of these transcription factors (ETS-1 log_2_FC = 2.54, *p* < 0.0001; TBX5 log_2_FC = −1.03, *p* < 0.0001).

Few genes encoding calcium channel subunits (*CACNA1S*, *CACNA1B*, and *CACNG6*; Figure 5F) were upregulated in FD hiPSC-VCMs compared with WT hiPSC-VCMs. Most transcripts of differentially expressed genes encoding calcium channel subunits were downregulated in FD hiPSC-VCMs compared with wild-type hiPSC-VCMs (*CACHD1*, *CACNA1H*, *CACNB2*, *CACA1C-AS1*, *CACNA1G-AS1*, and *CACNA1G*; Figure 5F). Furthermore, ryanodine receptor genes were also differentially expressed in FD hiPSC-VCMs compared with WT hiPSC-VCMs (RYR1 log_2_FC = 2.38, *p* < 0.0001; RYR2 log_2_FC = −0.7, *p* < 0.0001; RYR3 log_2_FC = −1.64, *p* < 0.0001). Restoration of GLA expression in adv-GLA FD hiPSC-VCMs increased the number of differentially expressed genes encoding calcium channel subunits from 9 to 12 compared with WT hiPSC-CMs (*CACNA1H*, *CACNA2D1*, *CACHD1*, *CACNB2*, *CACNA1G-AS1*, and *CACNA1G* were downregulated, whereas *CACNAG8*, *CACNA1A*, *CACNAG7*, *CACNAG6*, *CACFD1*, and *CACNA1B* were upregulated; Figure 5F). Additionally, overexpression of GLA in adv-GLA hiPSC-VCMs increased the expression of RYR1 (log_2_FC = 3.76, *p* < 0.0001) and decreased the expression of RYR2 (log_2_FC = −1.05, *p* < 0.0001) but equalized the expression of RYR3 relative to WT hiPSC-VCMs.

Transcription factors known to control the expression of genes encoding calcium channels, including members of the TBX family, were dysregulated in FD hiPSC-VCMs compared with WT hiPSC-VCMs (TBX2 log_2_FC = 2.89, *p* < 0.0001; TBX5 log_2_FC = −1.03, *p* < 0.0001; TBX18 log_2_FC = −2.89, *p* < 0.0001) and remained differentially expressed after adv-GLA treatment of FD hiPSC-VCMs (TBX2 log_2_FC = 2.87, *p* < 0.0001; TBX5 log_2_FC = −1.03, *p* < 0.0001; TBX18 log_2_FC = −2.87, *p* < 0.0001; TBX20 log_2_FC = −1.3, *p* < 0.0001).

## Discussion

This study reports three significant findings. First, a new approach methodology for FD cardiac pathophysiology modeling was developed by reprogramming fibroblasts from patients carrying *GLA* variants into hiPSCs and differentiating these hiPSCs into ventricular cardiomyocytes. Second, applying this new approach methodology, we observed that hiPSC-VCMs exhibited intrinsic defects in action potentials that phenocopied the severe arrhythmias commonly observed in FD patients. Third, most of the observed conduction abnormalities were not reversed by restoration of GLA expression.

Functional deficiency of GLA is well known to cause accumulation of Gb_3_ in lysosomes (8, 9) of cells from FD patients, including myocardial cells (28, 29). We used dermal fibroblasts from three different patients carrying mutations reported by the cell bank (Corriell, USA) to lack GLA activity (GM00881) or to retain 10% (GM02771) and 50% (GM00107) of the enzyme activity. Irrespective of the reported GLA activity, all fibroblast lines exhibited significant accumulation of Gb_3_ compared with WT cells. *GLA* mutations and Gb_3_ accumulation did not affect the ability to reprogram fibroblasts into hiPSCs. hiPSC lines were derived from all three fibroblast lines. However, two of the hiPSC lines (GM02771 and GM00107) displayed abnormal karyotypes unrelated to reprogramming with the Yamanaka factors, as these aneuploidies were already present in the dermal fibroblasts obtained from the cell bank. These findings support the need to include fibroblast karyotyping as the first step in generating hiPSCs from donors and cell banks to establish a reliable NAM. Due to the similarities in Gb_3_ accumulation between the three fibroblast lines and the presence of aneuploidy in two fibroblast lines, subsequent electrophysiology functional studies of FD hiPSC-VCM were conducted using derivatives of the GM00881 fibroblast line.

Cardiac symptoms of FD are clinically significant due to the high incidence of ventricular arrhythmias, atrial fibrillation, and sudden cardiac death, which may account for up to 62% of the deaths in FD patients, even when receiving treatment (48). Therefore, understanding the structural and functional characteristics of cardiomyocytes derived from Fabry disease patients using a new approach methodology that yields relevant cardiobiological data is essential for tailoring therapeutic strategies. New approach methodologies employing hiPSCs from FD patients have been employed to study the effects of substrate reduction strategies (49), to develop FD models for high-throughput screening of drugs (50), to evaluate the efficacy of modified messenger RNA (modRNA) (51) therapies on Gb_3_ accumulation, and to assess the effects of FD on the cardiomyocyte proteome (52). However, these studies did not focus on characterizing the action potentials and intracellular calcium handling of FD cardiomyocytes.

To address the lack of knowledge about the functional properties of FD cardiomyocytes, we differentiated FD hiPSCs into ventricular cardiomyocytes using a well-characterized protocol previously described (34–36). Cardiomyocytes were purified using magnetic-assisted cell sorting (37) and subsequently plated to form functional syncytia of matured cardiomyocytes (34, 35, 39), which were used for optical mapping of action potentials or intracellular calcium handling, assessment of adenovirus-mediated GLA rescue, and transcriptomic analysis.

The initial assessment of action potentials in mature syncytia of FD hiPSC-VCMs using optical mapping indicated polymorphic arrhythmias with severe alterations in beat intervals, including tachyarrhythmias, bradyarrhythmias, and sudden quiescence. Routinely, optical mapping acquisition time is limited to 10 s, as reported for WT hiPSC-VCMs; however, in this study, the acquisition time was extended to 30 s for FD hiPSC-VCMs to capture the complex nature of FD-associated arrhythmias. The FD hiPSC-VCM model elicited *in vitro* polymorphic ventricular arrhythmias that deteriorated to Torsades de Pointes in five out of 16 functional syncytia of FD hiPSC-VCMs (Figure 2). Furthermore, despite the higher frequency of spontaneous activation and shorter Fridericia-corrected APD_80%_, FD hiPSC-VCMs showed prolonged APD_tri_, corresponding to phase 3 of action potential repolarization. This finding is indicative of aberrations in potassium currents and represents a potent predictor of arrhythmias (53). In addition, conduction velocity was reduced in FD hiPSC-VCMs. Reduction in conduction velocity and prolongation of APDtri have long been recognized as important contributors to the development of arrhythmias (53–57). These findings suggest that the FD hiPSC-derived ventricular cardiomyocyte model recapitulates the *in vitro* arrhythmic phenotype observed in FD patients, which is characterized by a high incidence of ventricular tachyarrhythmias and bradyarrhythmias (58).

Bradyarrhythmias were observed in FD hiPSC-VCMs compared with WT hiPSC-VCMs during optical mapping of intracellular calcium transients. This finding may reflect the polymorphic nature of ventricular arrhythmias in patients with FD, in whom shifts between bradyarrhythmias and tachyarrhythmias are observed. Although autonomic dysfunction and chronotropic incompetence have been proposed as mechanisms underlying bradyarrhythmias in FD, hiPSC-CM models of FD, including ours, do not receive input from the autonomic nervous system (49–52), which suggests that while the autonomic nervous system may contribute to the initiation of arrhythmias in FD, the underlying electrical alterations are intrinsic to FD cardiomyocytes. Furthermore, we tested the chronotropic competence of these cells by stimulating the β-adrenergic system by treating FD hiPSC-VCMs syncytia with isoproterenol (100 nM). For this experiment, we opted to perform optical mapping of intracellular calcium transients to better evaluate important aspects of cardiomyocyte responses to β-adrenergic stimulation, including chronotropic (increased frequency of contraction), ionotropic (increased amplitude of calcium fluorescence), and lusitropic (changes in CaTD_80_ and CaTD_tri_) effects.

Isoproterenol has been reported as a tool to reverse prolonged pauses in heartbeat and complete heart block (59), as well as a probe to investigate dual AV node physiology (60) in FD patients. Interestingly, β-adrenergic stimulation was also able to restore rhythmic activity in the FD hiPSC-VCM model. Furthermore, the relative change in spontaneous frequency of contraction induced by isoproterenol was greater in FD hiPSC-VCMs compared with WT hiPSC-VCMs. The relative changes induced by isoproterenol in the amplitude of calcium fluorescence, CaTD_80_, and CaTD_tri_ were comparable between WT and FD hiPSC-VCMs, indicating that FD hiPSC-VCMs retain preserved chronotropic, inotropic, and lusitropic responses.

Although FD hiPSC-VCMs exhibited a preserved β-adrenergic response, the frequency of spontaneous contraction in isoproterenol-treated FD hiPSC-VCMs was significantly lower than in WT hiPSC-VCMs, both with and without β-adrenergic stimulation. As expected, absolute values of CaTD_80_ and CaTD_tri_ were prolonged in FD hiPSC-VCMs compared with WT hiPSC-VCMs, owing to the lower activation frequency of cardiomyocytes. Interestingly, baseline calcium fluorescence, which serves as a surrogate for diastolic intracellular calcium levels, was significantly reduced in DF hiPSC-VCMs compared with WT cardiomyocytes; however, the amplitude of intracellular calcium transients was significantly higher in FD hiPSC-VCMs, consistent with a previous report showing augmented intracellular calcium amplitude in FD cardiomyocytes (52).

ERT has shown minimal improvement in the cardiac phenotype of FD patients, despite reducing Gb_3_ accumulation and improving other systems (61). More specifically, patients presenting with ventricular hypertrophy may experience a slight decrease in ventricular mass (61); however, arrhythmias present before the initiation of treatment often persist after ERT or may develop during an ERT regimen (62). Substrate reduction and GLA modRNA strategies have been demonstrated to decrease the accumulation of Gb_3_ in cardiomyocytes derived from FD hiPSCs (49, 51). We tested the impact of Gb_3_ clearance through adenovirus-mediated expression of GLA on the abnormal contractility of FD hiPSC-VCMs. Overexpression of GLA decreased the accumulation of Gb_3_ in FD hiPSC-VCMs; however, it did not significantly change the percentage of matured FD hiPSC-VCM syncytia exhibiting arrhythmias. Disturbingly, GLA overexpression induced tachyarrhythmias in 30% of FD hiPSC-CMs, increasing the arrhythmic burden to 52% (arrhythmias plus tachyarrhythmias). These findings are consistent with clinical data indicating that arrhythmias may occur under conditions that reduce Gb_3_ accumulation (62). Despite the increased arrhythmia burden, GLA overexpression produced significant changes in intracellular calcium handling in FD hiPSC-VCMs, as indicated by an increase in the frequency of spontaneous contractions and decreases in CaTD_80_, CaTD_tri_, and calcium fluorescence amplitude.

To better understand the impact of GLA overexpression in FD hiPSC-VCMs, we performed poly(A)-enriched mRNA sequencing. GO analysis revealed that calcium ion binding (GO:0005509) was among the molecular functions altered in FD compared with WT hiPSC-VCMs. Other GO-annotated molecular functions differentially expressed in FD relative to WT hiPSC-VCMs included signaling receptor binding (GO:0005102) and molecular transducer activity (GO:0060089). Overexpression of GLA in adv-GLA FD hiPSC-VCMs further altered the GO molecular functions associated with voltage-gated sodium channel activity involved in cardiac muscle cell action potential (GO:0086006) and channel activity (GO:0015267) compared with FD hiPSC-VCMs. The increase in GO:0086006 might explain the appearance of tachyarrhythmias in adv-GLA FD hiPSC-VCMs, as sodium influx into cardiomyocytes may elevate the resting membrane potential closer to the activation threshold (52). In comparison with WT hiPSC-VCMs, adv-GLA FD hiPSC-VCMs exhibited GO molecular functions associated with cytoskeletal binding (GO:0008092 and GO:0003779), which may have important implications for cell physiology and even electrophysiology, as macromolecular complex trafficking is an important process that can alter cardiomyocyte action potential (9, 63).

We performed a more in-depth analysis of the transcriptomic profile of genes encoding subunits of sodium, potassium, and calcium channels. Overall, transcripts of genes encoding these ion channel subunits were differentially expressed between FD and WT hiPSC-VCMs. FD hiPSC-VCMs showed reduced expression of genes encoding the alpha subunits (*SCN1A*, *SCN5A*, and *SCN9A*) of sodium voltage-gated channels and beta subunits of this channel (*SCN2B* and *SCN4B*) compared with WT hiPSC-VCMs. Mutations in *SCN5A*, *SCN1A*, *SCN2B*, and *SCN4B* have been reported to associate with different arrhythmic conditions, such as Brugada syndrome, atrial fibrillation, long QT syndrome type 3, and sudden infant cardiac death (64–74). Interestingly, *SCN9A* is canonically recognized as encoding a sodium channel subunit involved in nociception, and mutations in this gene are associated with pain disorders (75–79); however, limited reports suggest that alterations in this gene may also be associated with arrhythmias (80). Downregulation of these genes may be directly related to decreased expression of transcription factors such as TBX-5, which is known to regulate the expression of some genes encoding sodium channel subunits (45–47).

Recovery of GLA expression in adv-GLA FD hiPSC-VCMs partially altered the expression profile of sodium channel genes. Transcripts of *SCN5A*, *SCN1A*, *SCN4B*, and *SCN2B* remained downregulated in adv-GLA FD hiPSC-VCMs compared with WT hiPSC-VCMs; in contrast, *SCN4A*, *SCN11A*, *SCN1B*, and the gene encoding sodium channel modifier 1 (*SCNM1*) showed increased expression after adv-GLA treatment compared with WT hiPSC-VCMs. Interestingly, *SCN11A* has not been reported to be expressed in the heart (81, 82), although it shares the locus with *SCN5A* and *SCN10A* on chromosome 3 (83). The persistent dysregulation of sodium channel gene transcription may contribute to the persistence of arrhythmias and the appearance of tachyarrhythmias even after reduction of Gb_3_ accumulation.

More than 80 genes encode potassium channel subunits expressed in different cell types throughout the human body (84). In FD hiPSC-VCMs, 29 of these genes were differentially regulated compared with WT hiPSC-VCMs. Nineteen of the differentially expressed genes were downregulated in FD hiPSC-VCMs relative to WT, including genes encoding inward rectifier potassium (K_ir_x) channels such as *KCNJ4* and *KCNJ2*. K_ir_x channels, such as K_ir_2.1 (encoded by *KCNJ2*), play an important role in modulating action potential duration and arrhythmogenesis (85–90). In the FD hiPSC-VCM model, the prolongation of APD_tri_ may be directly related to reduced transcription of genes encoding K_ir_x. Overexpression of GLA did not equalize potassium channel gene expression in adv-GLA FD hiPSC-VCMs relative to WT. In fact, after GLA overexpression, 32 genes encoding potassium channels were differentially expressed, and *KCNJ2* remained downregulated, along with other K_ir_x genes such as *KCNJ3*, *KCNJ4*, *KCNJ5*, *KCNJ8*, and *KCNJ11*.

Genes encoding calcium channels were also differentially expressed in FD hiPSC-VCMs compared with WT. Three genes were upregulated, including those encoding the α1S subunit of the voltage-gated calcium channel Ca_v_1.1 (*CACNA1S*), the α1B subunit of Ca_v_2.2, and the gamma-6 subunit (*CACNG6*), while six genes were downregulated (*CACHD1*, *CACNA1H*, *CACNB2*, *CACA1C-AS1*, *CACNA1G-AS1,* and *CACNA1G*) relative to WT. Ca_v_1.1 is a component of the L-type calcium channel (91); however, this subunit has been reported to be enriched in skeletal muscle (92, 93), where it activates skeletal RYR1, which is overexpressed in FD hiPSC-VCMs. RYR1 has been reported to exhibit heterogeneous activation (94) and is susceptible to inactivation at 1 mM Ca^2+^, whereas RYR2 and RYR3 remain active at this concentration (94–98). RYR1 is mainly activated by membrane depolarization and subsequent activation of Ca_v_1.1, while RYR2 mainly responds to changes in intracellular calcium concentration (99). Furthermore, skeletal RYR (RYR1), which is upregulated in FD hiPSC-VCMs, exhibits high and fast Ca^2+^ conductance for a fast contractile response, and cardiac RYR (RYR2), which is downregulated in FD hiPSC-VCMs, shows moderate Ca^2+^ conductance. The ectopic overexpression of Ca_v_1.1/RYR1 and the downregulation of other Ca_v_ and RYR encoding genes might be important contributors to part of the phenotype observed in FD hiPSC-VCMs, characterized by a significantly increased upstroke slope and amplitude of intracellular calcium release.

In adv-GLA hiPSC-VCMs, the number of differentially expressed genes increased from 9 to 12. Although transcription of CACNA1S in adv-GLA FD hiPSC-VCMs was restored to levels comparable to WT, there was a significant increase in the expression of RYR1, a partner of Ca_v_1.1. These dysregulations in calcium channel gene expression may be directly related to alterated transcription of proteins involved in the transcription of calcium channels, such as TBX2, TBX5, and TBX8. These alterations in TBX2, TBX5, and TBX8 were not corrected by adv-GLA treatment.

In conclusion, the hiPSC-VCM model of Fabry disease recapitulates the complex arrhythmic phenotype observed in FD patients. Furthermore, transcriptomic analysis indicates that these arrhythmias may arise from widespread dysregulation of genes encoding different subunits of sodium, potassium, and calcium channels, and this dysregulation is resistant to GLA overexpression and reduction of Gb_3_ accumulation. Our report indicates that FD represents a high complexity channelopathy, which may be difficult to manage pharmacologically due to its multifactorial nature.

There are significant opportunities for future studies utilizing the hiPSC-VCM NAM described in this report. These include the generation of hiPSC-VCMs expressing different *GLA* variants, which may differ in genotypes and arrhythmia-associated phenotypes. The hiPSC-VCM model could be used to screen candidate anti-arrhythmic drugs for preventive therapy. Finally, the hiPSC-VCM model may provide deeper fundamental insights into the cardiac channelopathies underlying the fatal arrhythmias observed in FD. In the absence of such work, the use of implantable cardioverter–defibrillators for the prevention of fatal arrhythmias might be considered even in patients receiving ERT or other therapies aimed at reducing Gb_3_ accumulation.

## Data Availability

The mRNA data presented in the study are deposited in the Gene Expression Omnibus (GEO, NIH) repository, accession number GSE327944.
