# Supplementary material for: A new approach methodology for studying intrinsic ventricular arrhythmias in Fabry disease
Source: Front Cardiovasc Med. 2026 Apr 23;13:1769383. doi: 10.3389/fcvm.2026.1769383 (PMC13149172; doi:10.3389/fcvm.2026.1769383)
Supplement: Supplementary file 1 [file Supplementaryfile1.pptx]

## Slide 1
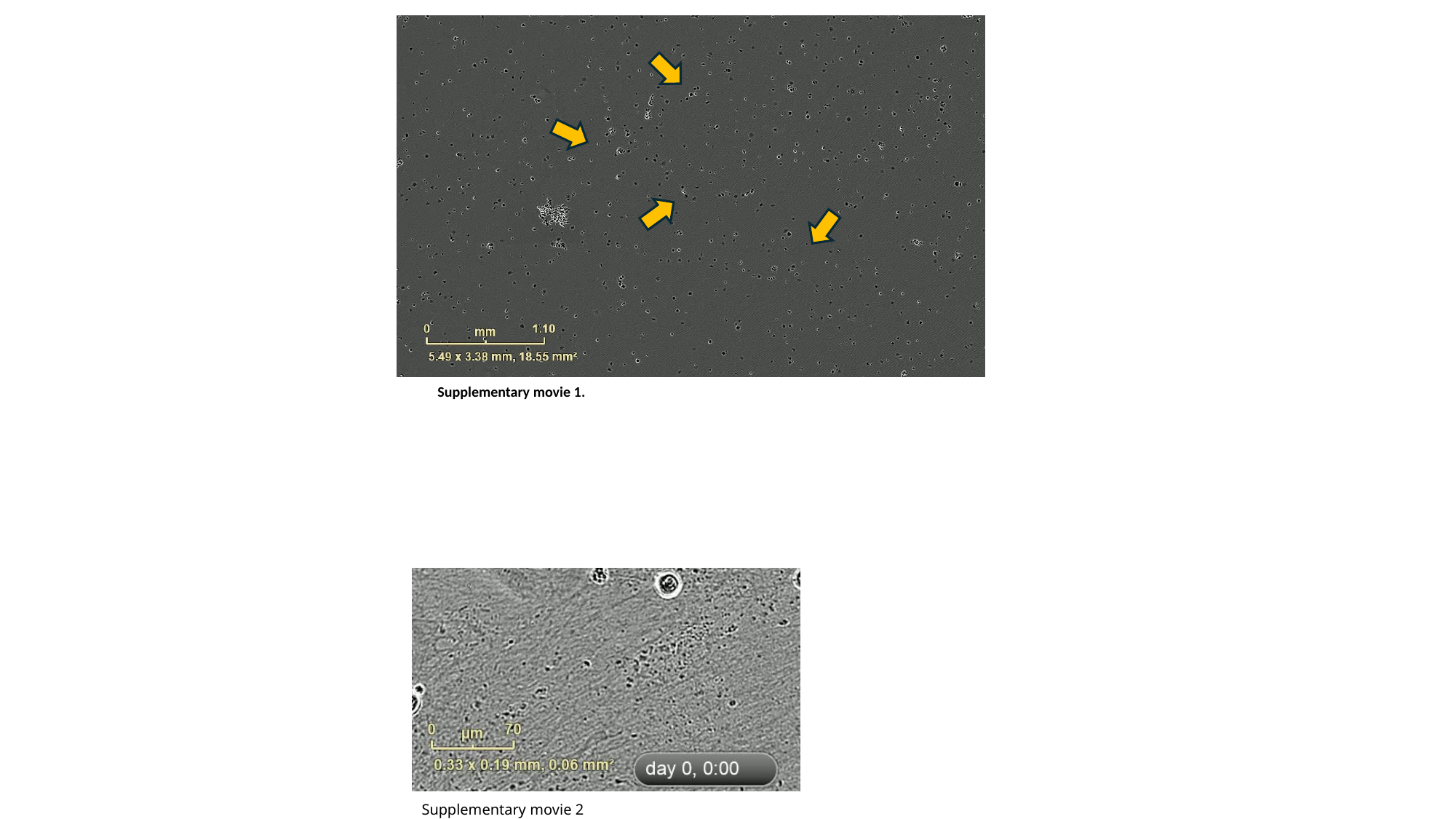

Supplementary movie 1.
Supplementary movie 2

## Slide 2
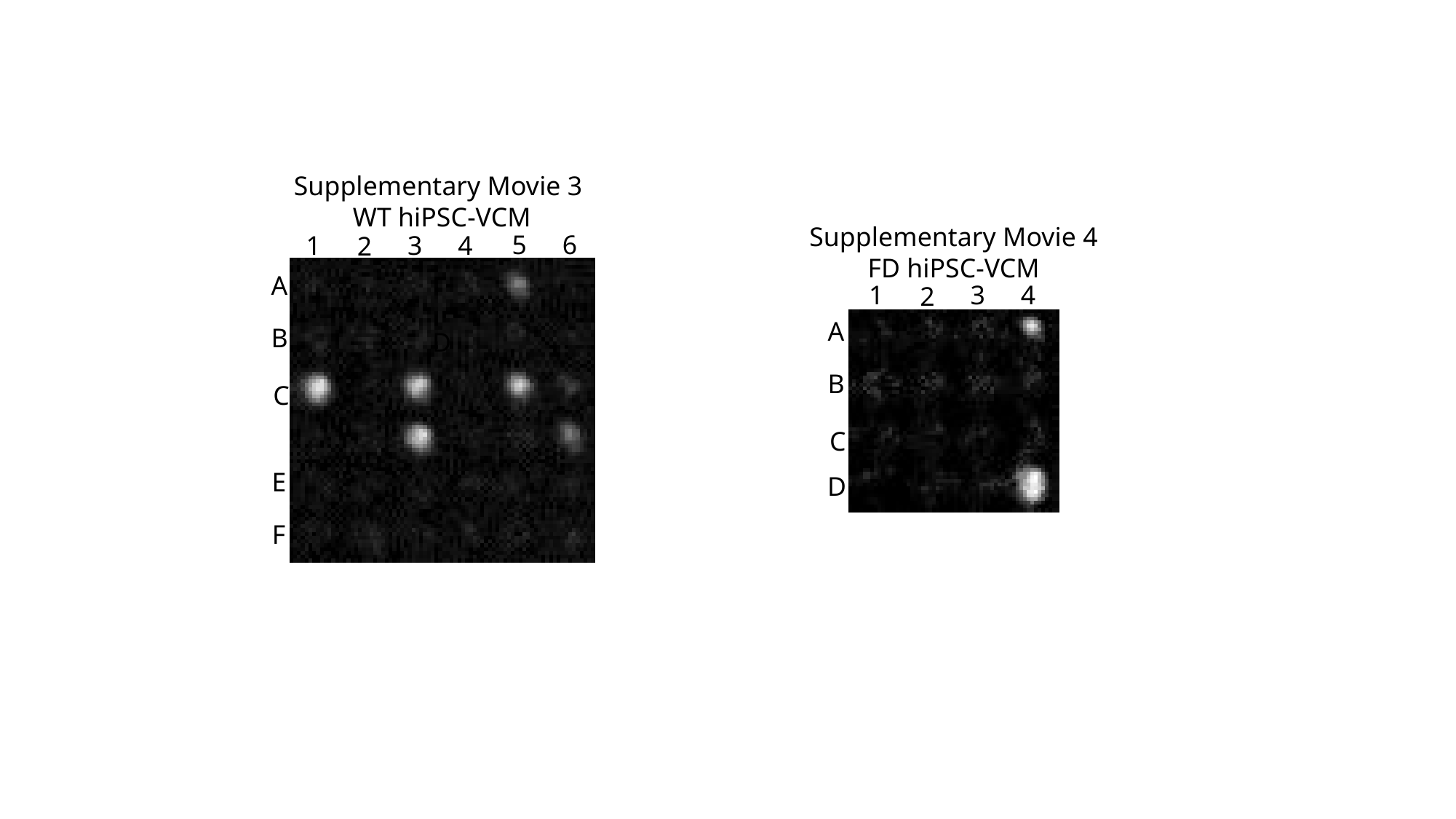

Supplementary Movie 3
 WT hiPSC-VCM
5
6
1
3
4
2
A
B
C
E
F
Supplementary Movie 4
FD hiPSC-VCM
1
3
4
2
A
B
C
D
D
