# Supplementary material for: A new approach methodology for studying intrinsic ventricular arrhythmias in Fabry disease
Source: Front Cardiovasc Med. 2026 Apr 23;13:1769383. doi: 10.3389/fcvm.2026.1769383 (PMC13149172; doi:10.3389/fcvm.2026.1769383)

## Slide 1
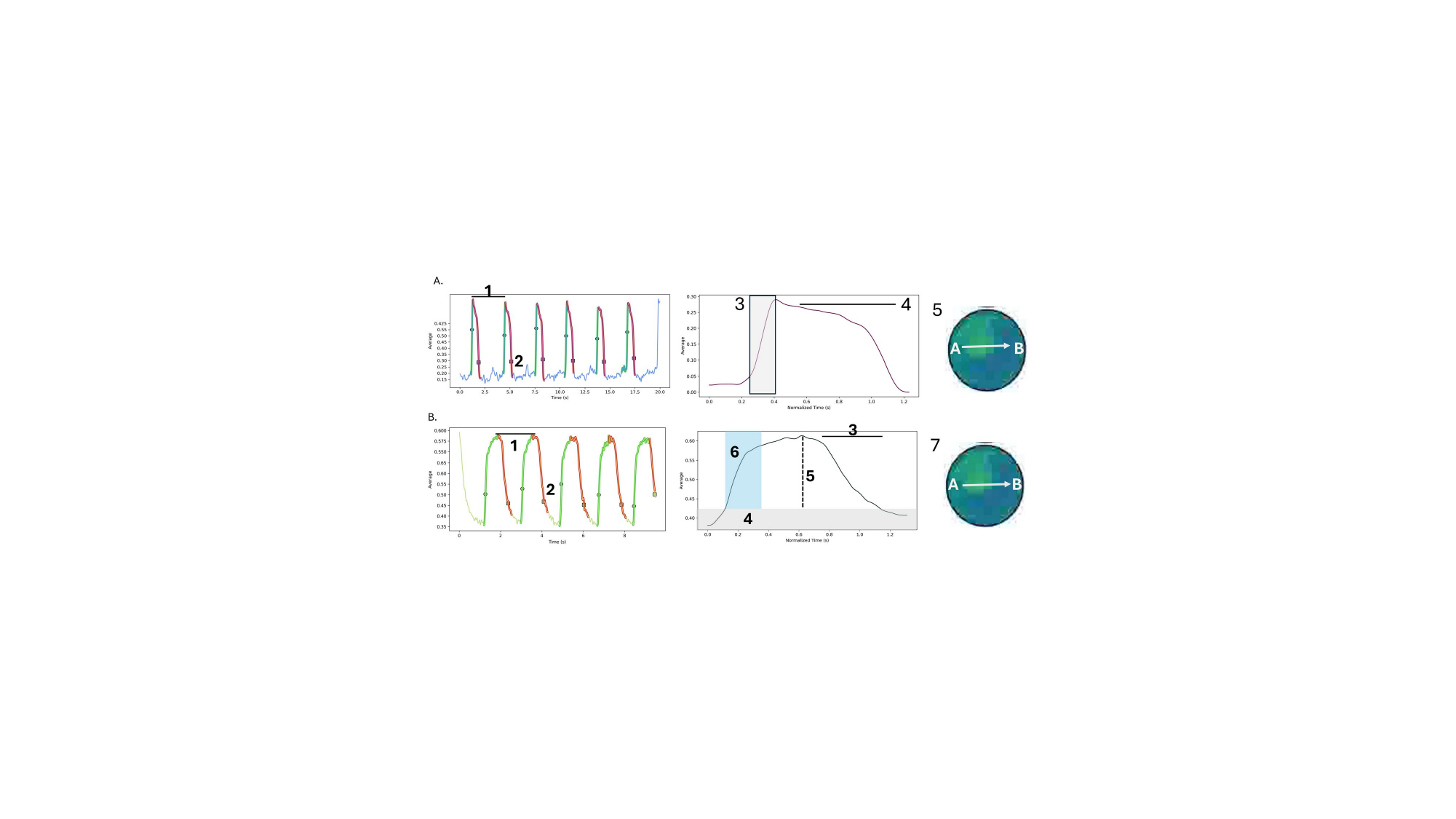

## Slide 2
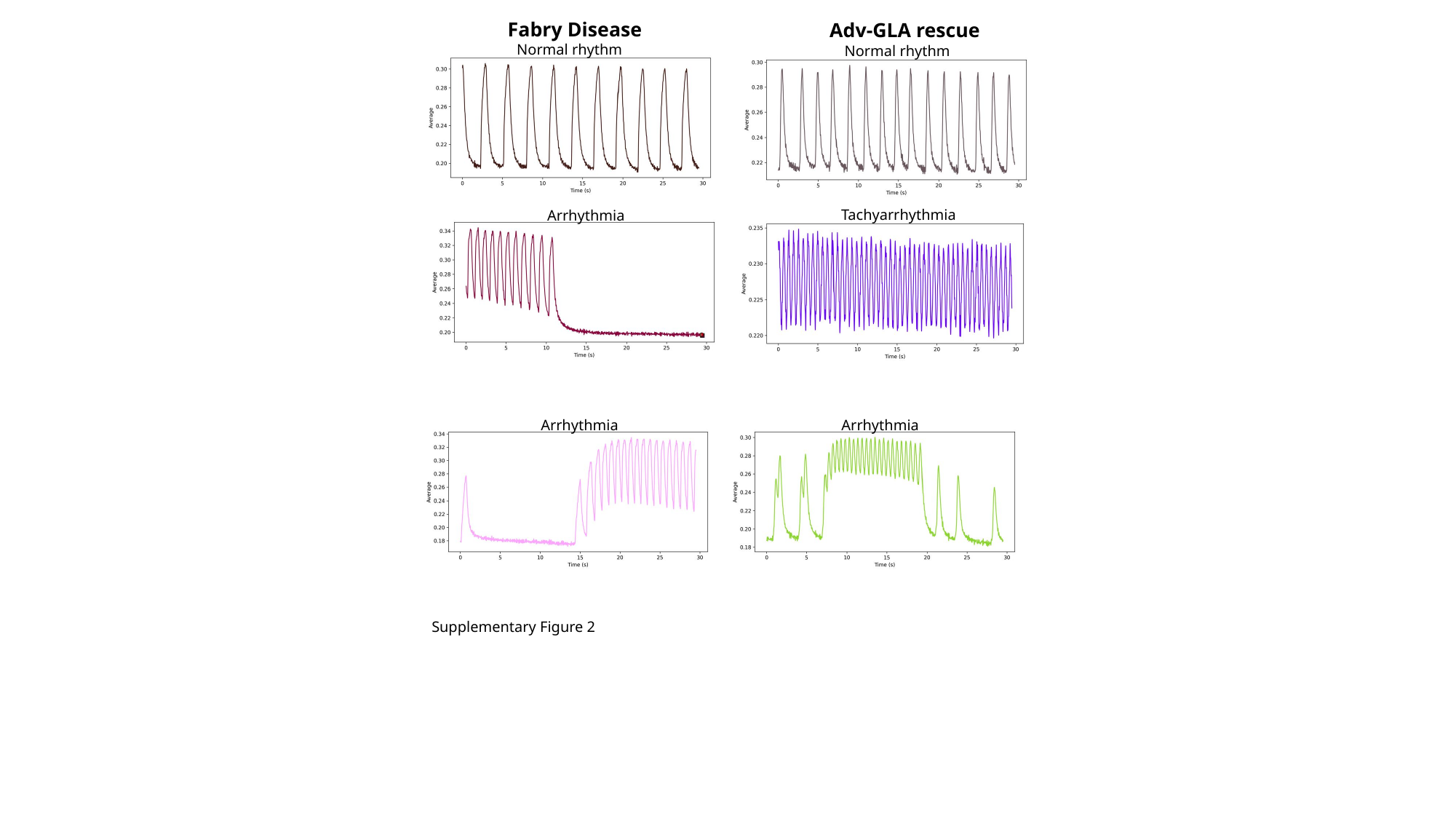

Fabry Disease
Adv-GLA rescue
Normal rhythm
Normal rhythm
Tachyarrhythmia
Arrhythmia
Arrhythmia
Arrhythmia
Supplementary Figure 2

## Slide 3
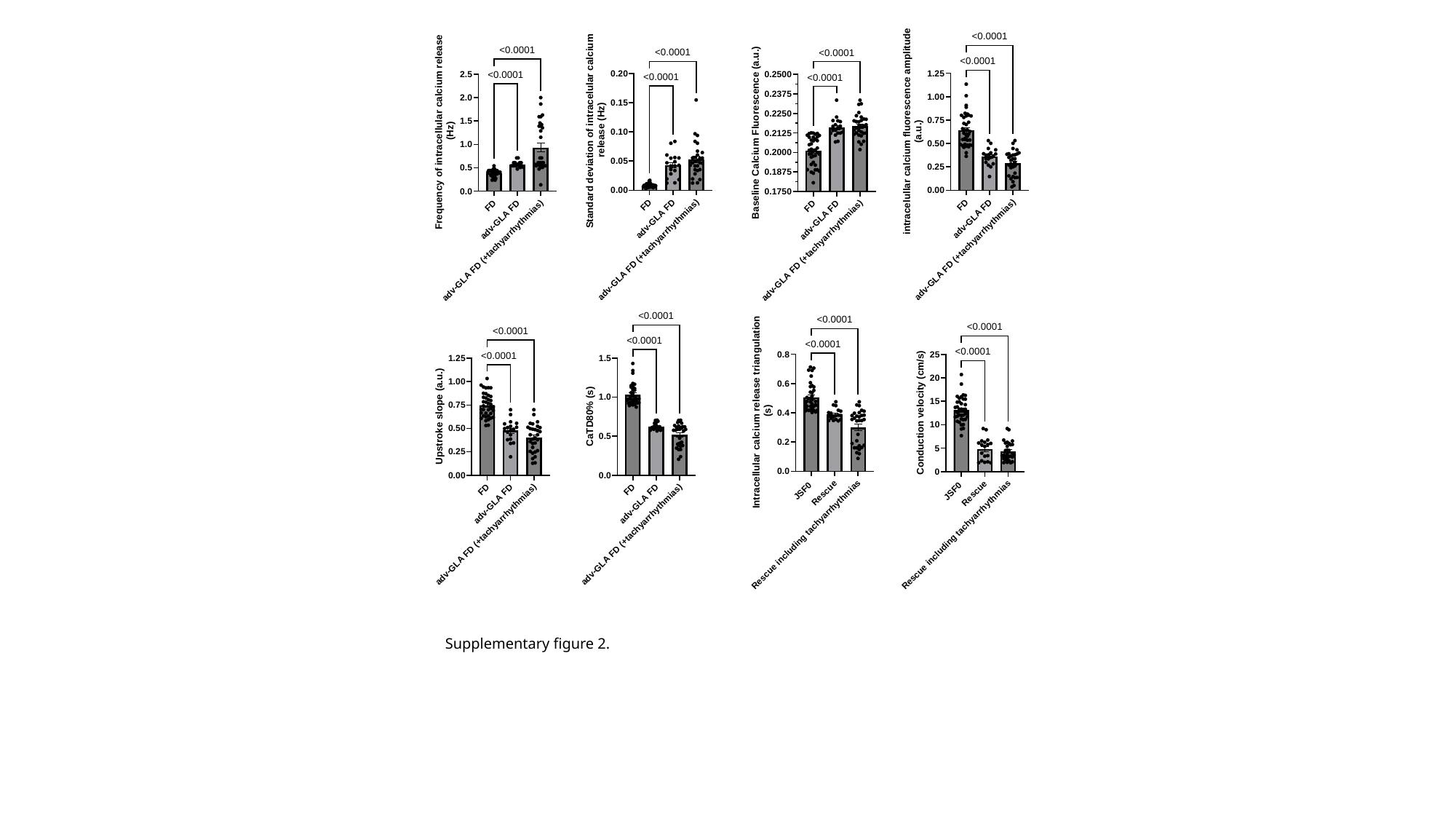

Supplementary figure 2.

## Slide 4
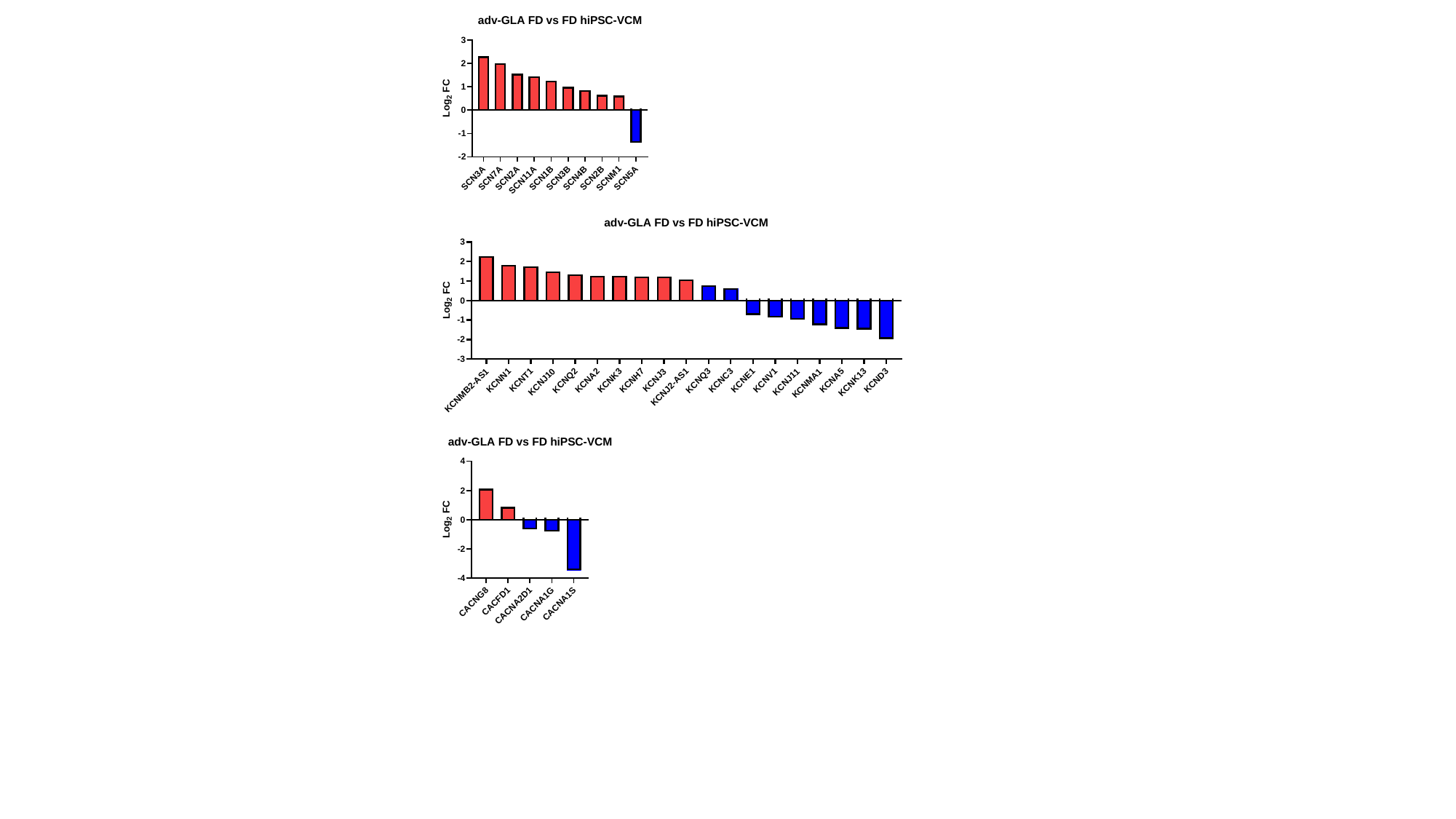

Supplement: Supplementary file 2 [file Presentation1.pptx]
